# A varying-coefficient cox model for the effect of CA19-9 kinetics on overall survival in patients with advanced pancreatic cancer

**DOI:** 10.18632/oncotarget.15557

**Published:** 2017-02-21

**Authors:** Yuntao Chen, Zhenyi Shao, Wen Chen, Hua Xie, Zhenyu Wu, Guoyou Qin, Naiqing Zhao

**Affiliations:** ^1^ Department of Biostatistics, School of Public Health and Key Laboratory of Public Health Safety, Fudan University, Shanghai 200032, China; ^2^ Collaborative Innovation Center of Social Risks Governance in Health, Fudan University, Shanghai 200032, China

**Keywords:** CA19-9, pancreatic cancer, varying coefficient model, biomarker, prognosis

## Abstract

**Purpose:**

To evaluate the relationship between serum CA19-9 and overall survival in patients with advanced pancreatic cancer.

**Methods:**

109 advanced pancreatic cancer patients with gemcitabine based first-line chemotherapy were included. The effect of pretreatment CA19-9 level on overall survival was modeled by Cox proportional hazard regression. The effect of CA19-9 kinetics on overall survival was modeled by an extended Cox regression with a time varying coefficient and a time varying covariate.

**Results:**

Univariate analysis indicated that baseline CA19-9 correlated with OS (HR = 1.66, *p* < 0.01) and this association remained significant within multivariate analysis (HR = 1.56, *P* < 0.01). For the analysis of CA19-9 kinetics, the extended Cox model showed that the effect of CA19-9 on overall survival changed with time: increased in the first two months and reached the top at a HR of about 2, then decreased for the next two months to a HR of about 1.56 and finally tended to be stable. The combination of pretreatment CA19-9 and CA19-9 at 2 month may better evaluate the patients’ prognosis compared to pretreatment CA19-9 alone.

**Conclusion:**

Pretreatment CA19-9 and CA19-9 kinetics may serve as a useful serum biomarker in advanced pancreatic cancer.

## INTRODUCTION

Pancreatic cancer (PC) is associated with a very poor prognosis, highlighted by the close parallel between disease incidence and mortality [1]. More than 80% of patients are initially diagnosed in an advanced stage of disease, where the potential curative resection is no longer possible. Gemcitabine is still being regarded as the standard chemotherapy for the treatment of locally advanced and metastatic disease [2]. However, treatment effects remain moderate with median overall survival (OS) times in the range of 5 to 8 months and 1-year survival rates in the range of 17–25% [3]. Therefore, in addition to a good therapeutic option, establishing clinically relevant prognostic biomarkers is also very important for this aggressive disease.

More than 2000 studies of biomarkers in pancreatic cancer has been published, implicating more than 2000 different genes and proteins [4]. Many studies concerning the correlation of SMAD4 genetic and molecular alteration with prognosis has been conducted. PC patients with SMAD4 expression had significantly longer survival as compared to those lacking SMAD4 expression [5]. Circulating tumor cells and circulating tumor DNA constitute easily accessible blood-borne tumor biomarkers that may prove their clinical interest for screening, early diagnosis and metastatic risk assessment of PC [6]. However, none of these biomarkers have been showed to possess the requisite sensitivity/specificity to be introduced in clinical use. The carbohydrate antigen 19-9 (CA19-9) is still the most commonly used and best validated serum tumor marker for pancreatic cancer diagnosis in symptomatic patients and for monitoring therapy in patients with pancreatic adenocarcinoma [7].

CA19-9 was first discovered in 1979 by researchers using monoclonal antibodies to isolate tumor associated antigens in colorectal carcinoma and two years later was also found to be produced by pancreatic carcinoma [8, 9]. However, approximately 5% to 14% of the population is Lewis antigen A and B negative (Le^a-b-^), and is also considered CA19-9 nonsecretory (CA19-9 < 5U/mL), which is correlated with poor survival [10]. Therefore these patients are excluded from this study. Since accurate determination of treatment response by imaging often remains difficult (e.g., due to the desmoplastic stroma reaction induced by the tumor itself in surrounding soft tissue), the serum tumor marker CA19-9 has been studied for several years if it could serve as an appropriate surrogate parameter of treatment efficacy [11].

Several previous studies suggested a significant correlation between serum CA19-9 and survival end point in patients receiving systemic chemotherapy for advanced pancreatic cancer [12–19]. Most of these studies discussed the relation between the pretreatment CA19-9 and OS [12, 13]. Some studies still considered a constant effect of CA19-9 on OS though a CA19-9 kinetics analysis was taken. [18–21] It was reported that the value of individual prognostic factors may change dependent on the length of the follow-up time; for example, the effect of a treatment can be strong immediately after treatment but fades with time [22]. Using Cox proportional hazards (PH) regression when the PH assumption was violated may produce biased results [23]. Time dependency has been accounted for and reported in oncology publications, such as in breast, colon and gastric cancer studies [24–28]. But it has not been considered in a pancreatic cancer study before. In addition, these published studies were limited to fixed covariates, measured at time of diagnosis. In this study, an extended cox model with a time varying coefficient and a time varying covariate was used to solve the problem that the effect of CA19-9 on OS was changing and a serial CA19-9 measurements should be included in the model.

The aims of this retrospective study were as follows: first, to evaluate the prognostic role of pretreatment CA19-9 in patients with advanced pancreatic cancer. Second, to construct an extended cox model which can consider not only the dynamic change of CA19-9 during the treatment, but also the different effect of CA19-9 on OS in the course of the treatment. Last, according to the above knowledge about the entire path variation of CA19-9's effect on OS during chemotherapy, to better predict the PC patients’ prognosis in combination with the pretreatment CA19-9.

## RESULTS

### Patient characteristics

Basic characteristics of 109 PC patients included in this study were listed in Table 1. The mean of age before chemotherapy for all patients was 64 years, ranging from 39 to 86 years. Males and females were almost equivalent in number (59 vs 50). Tumors were more likely to occur at the head or neck of pancreas (n = 50, 46%) than the body or tail (n = 35, 32%). The tumor location of the remaining 24 cases couldn't be figured out due to the insufficient diagnostic information (diagnosis such as pancreatic carcinoma *in situ* or pancreatic malignant tumor). The majority of patients had metastasis before the chemotherapy (n = 70, 64%), and 71% of which metastasized to liver with the remaining spreading to lung, bone, brain and so on. The median OS of the patients was 7.4 months (range, 1 - 34.1 months). The pretreatment CA19-9 measurements were done in the previous one month before the start of first-line chemotherapy. The median pretreatment CA19-9 in the study population was 532.5 U/mL (range, 5.1 - 10000 U/mL). 77% (84) of patients had 2 or more CA19-9 measurements during the chemotherapy (median, 3; range, 1-16). The median CA19-9 level during the chemotherapy was 823.9 U/mL (range, 5.3 - 12777 U/mL).

**Table 1:** Baseline patient characteristics (n=109)

	No.	%
Gender		
Male	59	54.13
Female	50	45.87
Chemotherapy		
Gemcitabine alone	41	37.61
Gemcitabine combined with other drugs*	68	62.39
Primary pancreas tumor		
Head	50	45.87
Body or tail	35	32.11
Unknown	24	22.02
Stage of disease		
Locally advanced	10	9.17
Metastatic	70	64.22
Unknown	29	26.61
Distant metastasis		
Liver	50	71.43
Lung	2	2.86
bone	4	5.71
Abdomen	5	7.14

^*^ other drugs include oxaliplatin, erlotinib, capecitabine, or 5-Fluorouracil. Most of the combination are gemcitabine and oxaliplatin.

### Pretreatment CA19-9

The pretreatment CA19-9 level was identified as a prognostic factor for OS in univariate and multivariate analyses, respectively. Every 9-fold CA19-9 increase means a HR of 1.66 (95% *CI*: 1.30 - 2.12, *P* < 0.01) in the univariate Cox regression model, and after adjusting for age, sex and chemotherapy (Gemcitabine combined with other drugs vs Gemcitabine alone), the HR for the effect of every 9-fold CA19-9 increase was estimated with 1.56 (95% *CI*: 1.20 - 2.01, *P* < 0.01). Residual analyses showed adequate fit of the multivariate Cox model especially with regard to the proportional hazards assumption and linearity assumption.

### Changeable effect with CA19-9 kinetics

**Figure 1:**
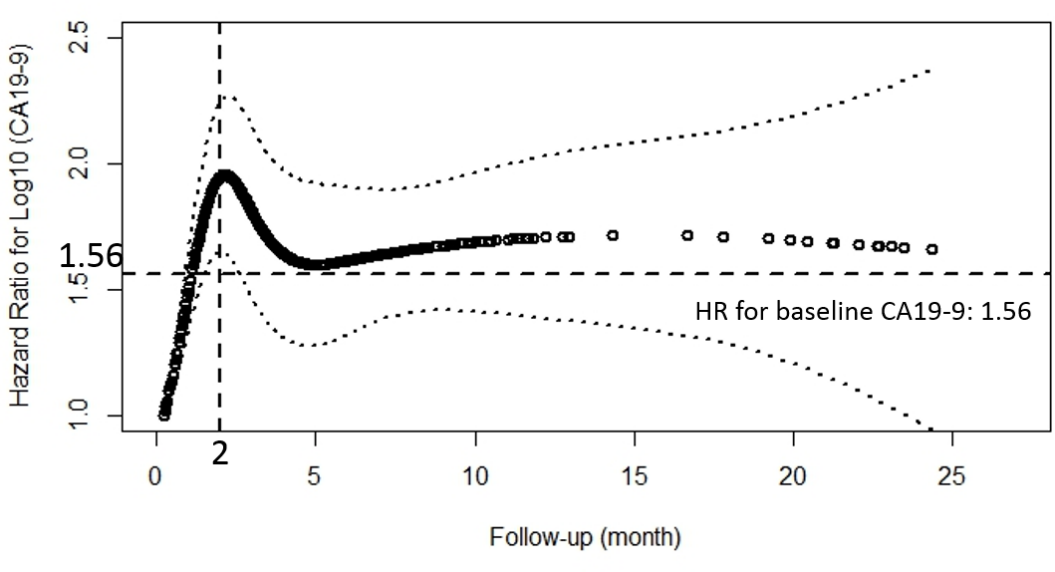
Estimates of the effect of peri-treatment CA19-9 on overall survival using the natural spline, presented as hazard ratio (solid line) and 95% CI (dashed lines) in extended Cox model with time-varying covariates and hazard ratio The horizontal reference line was set at 1.56, which was the HR for the effect of baseline CA19-9 on OS. The vertical reference line was set at 2 month, when HR for the effect of peri-treatmnet CA19-9 on OS reached the top.

Considering the varying effect of CA19-9 on OS, we firstly confirmed this effect by a schoenfeld residual plot.(Supplementary Figure 1) Though the test for PH assumption was not significant (*p* = 0.78), we thought it should be rejected for that a quadratic shape for β(t) might be apparent on the plot, but be undetected by the test for linear slope [29]. In order to get a more objective and comprehensive result, CA19-9 kinetics was applied in place of the CA19-9 measurement at one time point. Finally a Cox model with a time varying coefficient and a time varying covariate was constructed and the result showed that the effect of CA19-9 on OS changed with time: increased in the first two months and reached the top at a HR of about 2, then decreased for the next two months to a HR of about 1.56 and finally tended to be stable. (Figure 1)

### The combination of pretreatment CA19-9 and CA19-9 at two months better predicts PC patients’ prognosis

**Figure 2:**
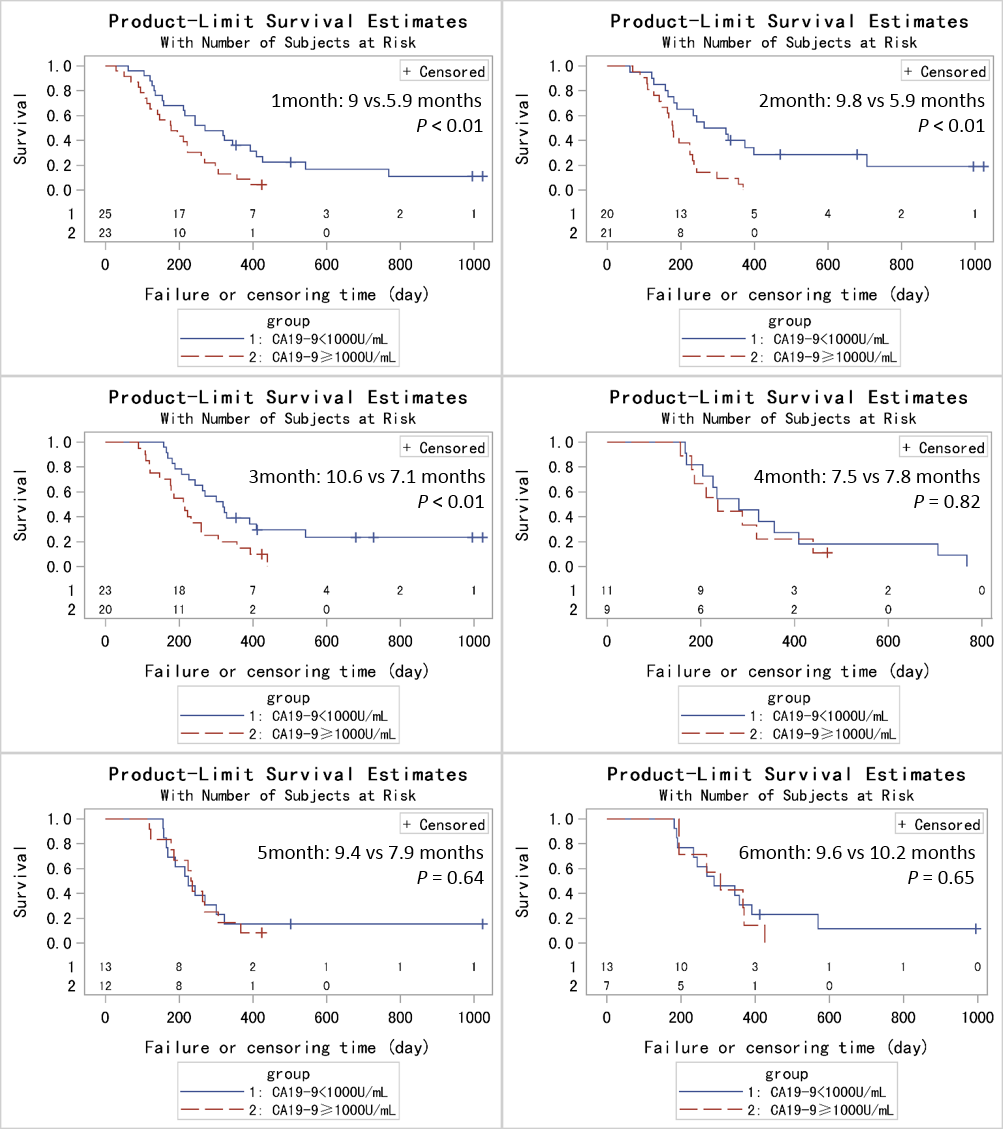
Kaplan-Metier analysis of OS for PC patients based on CA19-9 levels at different time points (1-6 months)

**Figure 3:**
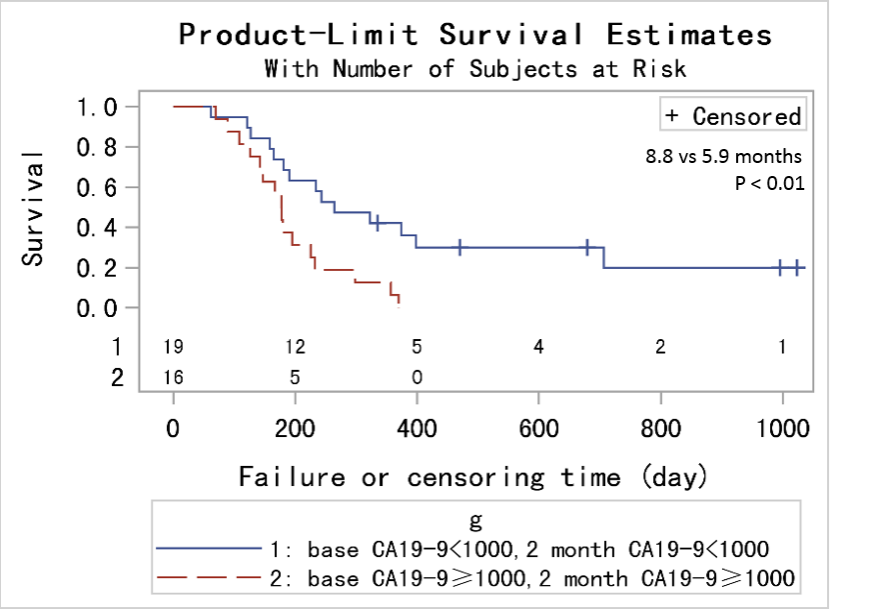
Kaplan-Metier analysis of OS for PC patients with pretreatment CA19-9 < 1000 U/mL and 2 month CA19-9 < 1000 U/mL compared to those with both ≥ 1000 U/mL

**Figure 4:**
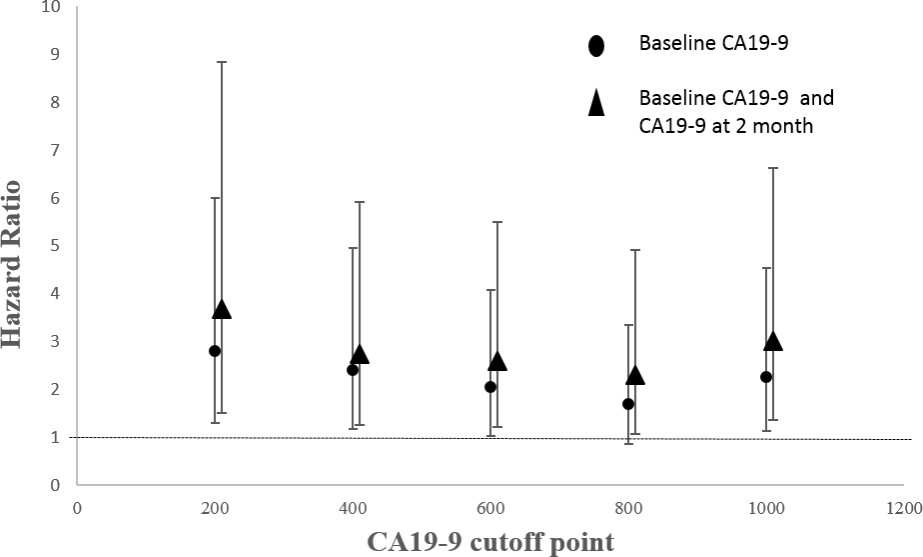
HRs with 95% CIs by different CA19-9 cutoff levels

In order to achieve a unique definition of (individualized and stage-adapted) cutoff points that will help to separate different prognostic subgroups, we divided the patients at different time points into two groups (≥ 1000 U/mL and < 1000 U/mL) according to the CA19-9 level [30]. We chose 1000 U/mL for two reasons. One was that previous studies took it [31], and the other was that many CA19-9 levels far larger than 1000 U/mL had been identified as 1000 U/mL in this database. Those patients with CA19-9 < 1000 U/mL had a longer median survival compared to those with CA19-9 levels ≥ 1000 U/mL at 1, 2, 3 months (9 *vs* 5.9 months, *p* < 0.01; 9.8 *vs* 5.9 months, *p* < 0.01; 10.6 *vs* 7.1 months, *p* = 0.01) using log-rank test while the results were not significant at 4, 5, 6 months (7.5 *vs* 7.8 months, *p* = 0.82; 9.4 *vs* 7.9 months, *p* = 0.64; 9.6 *vs* 10.2 months, *p* = 0.65). (Figure 2) After adjusting for age, pretreatment CA19-9, sex and chemotherapy, the HRs for the effect of CA19-9 (≥ 1000 *vs* < 1000 U/mL) on OS showed a tendency similar to Figure 1. Because of the limited sample size and adjustment of pretreatment CA19-9 in the model, we did not get a statistically significant result. HR reached the top at 2 month though the p value was not significant. (Table 2) Therefore, CA19-9 at 2 month might be the most influential prognostic factor in the course of chemotherapy. So the combination of pretreatment CA19-9 and 2 month CA19-9 was used to evaluate the patients’ prognosis, and those patients with pretreatment CA19-9 < 1000 U/mL and 2 month CA19-9 < 1000 U/mL had a longer median survival compared to those with both ≥ 1000 U/mL (8.8 *vs* 5.9 months, *p* < 0.01). (Figure 3) If pretreatment CA19-9 was considered alone, patients with pretreatment CA19-9 < 1000 U/mL had a longer median survival compared to those with pretreatment CA19-9 ≥ 1000 U/mL (8.0 *vs* 5.9 months, *p* = 0.02). Above results suggest that adding a 2 month CA19-9 do predict the prognosis of the subgroup with pretreatment CA19-9 <1000 U/mL more accurately though there is no difference in the prediction of the subgroup with pretreatment CA19-9 ≥ 1000 U/mL. Due to the fact that different clinical studies reported a wide discrepancy for a CA19-9 cutoff level (200-1212 U/ml) that makes identifying prognostic relevant subgroups possible [11], we also performed a sensitivity analysis based on different CA19-9 cutoff levels to check the robustness of the study results. As we can learn from Table 3 and Figure 4, the choice of cutoff levels did not influence the main conclusion.

**Table 2:** Impact of post-treatment CA19-9 levels (≥1000 vs <1000U/mL) on OS in a univariate and multivariate Cox model

Time	N	Univariate Cox model		Multivariate Cox model	
		Crude HR (95% CI)	*P*	Adjusted HR* (95% CI)	*P*
1 month	48	2.27(1.20-4.31)	0.01	0.56(0.03-11.77)	0.71
2 month	41	3.11(1.49-6.49)	<0.01	2.55(0.89-7.27)	0.08
3 month	43	2.34(1.19-4.58)	0.01	2.46(0.99-6.10)	0.05
4 month	25	1.21(0.52-2.81)	0.66	2.00(0.68-6.92)	0.21
5 month	20	1.26(0.48-3.28)	0.64	1.35(0.35-5.18)	0.66
6 month	20	1.25(0.47-3.31)	0.65	1.63(0.32-8.19)	0.56

^*^ adjust for age, baseline CA19-9, sex and chemotherapy.

**Table 3:** Prognostic value of the combination of pretreatment CA19-9 and CA19-9 at 2 month compared to the pretreatment CA19-9 alone

CA19-9 cutoff level, U/ml	Median survival, months	
	Pretreatment CA19-9^a^	Pretreatment CA19-9 and CA19-9 at 2 month^b^
1000	8.0 vs. 5.9 (p = 0.0171)	8.8 vs. 5.9 (p = 0.0043)
800	7.9 vs. 6.0 (p = 0.1141)	8.8 vs. 6.0 (p = 0.0273)
600	8.1 vs. 5.9 (p = 0.0344)	9.8 vs. 5.9 (p = 0.0114)
400	8.8 vs. 6.0 (p = 0.0138)	10.8 vs. 6.0 (p = 0.0079)
200	10.8 vs. 6.0 (p = 0.0060)	12.5 vs. 6.0 (p = 0.0022)

^a^ categorized by pretreatment CA19-9 alone (pretreatment CA19-9<m vs. pretreatment CA19-9≥m, m: CA19-9 cutoff level).

^b^ categorized by the combination of pretreatment CA19-9 and CA19-9 at 2 month (pretreatment CA19-9<m and CA19-9 at 2 month<m vs. pretreatment CA19-9≥m and CA19-9 at 2 month≥m, m: CA19-9 cutoff level).

## DISCUSSION

Detection of suitable predictive biomarkers to evaluate the treatment effect of chemotherapy and predict the prognosis is still a major challenge for advanced pancreatic cancer patients. CA19-9 is viewed as the most important serum biomarker in pancreatic cancer [32–34]. It greatly reflects tumor burden and activity more than any other marker that has been reported so far [33, 35, 36]. Though there were many studies confirming the association between the pretreatment CA19-9 level and OS [12, 13] and some investigators reported the effect of an entire path of CA19-9 levels on OS as well [18], to our knowledge a Cox model considering both varying effect of CA19-9 on OS and CA19-9 kinetics as a continuous variable has not been constructed yet to probe into the comprehensive relationship between CA19-9 and OS.

In our study, the method of Therneau and Grambsch (2000) [29] was used to expand the data set for the time dependent covariate CA19-9. And this method allowed us to make use of every CA19-9 measurement of one single patient, compared to the use of the pretreatment CA19-9 only, which may get a more precise determination of its prognostic biological significance. Meanwhile, a varying effect of CA19-9 kinetics on OS was also considered to get a more comprehensive result. The method to explore the varying effect of a time dependent covariate on outcome has been previously used in other researches. Bin et al. used it to investigate the effect of age at a marker event on age at menopause [37]. Robert et al. also took this method to explore the varying effect of race and maternal smoking on the risk of mortality post-LMP (last menstrual period) [38]. However, the previous studies all discussed a categorical and unordered time dependent variable, and to our knowledge, the present study is the first one to study the varying effect of an ordered time dependent variable.

Our study finally met its predefined end point: pretreatment CA19-9 level was found to be an independent prognostic factor for OS in univariate and multivariate analyses. The effect of CA19-9 levels on OS varied in the different course of the treatment, and more specifically the association between CA19-9 and OS became stronger with time for the first two months, and then weaker with time for the next two months, finally tended to be stable. Meanwhile the effect of the pretreatment CA19-9 level on OS was smaller than that of the post-treatment from the first to the fifth month. (Figure 1) Though at least 6 months are recommended for patients to receive chemotherapy, there are no RCT data to support duration of initial treatment. In practice, total duration of first-line chemotherapy is variable and dependent on patient tolerability and tumor response [39]. What's more, with current chemotherapy regimens, the median survival for patients with unresectable tumors is 9-11 months (this study: 7.4 months) [40]. Therefore, we can infer that patients usually receive first-line chemotherapy not longer than 6 months. One might conclude that adding a CA19-9 during the chemotherapy can better predict the prognosis compared to a pretreatment CA19-9 alone. The key problem is that there is no evidence about how long after the initial of treatment we should take the CA19-9 measurement though most of the studies consider 2 month as that time point [19, 41]. In this study we found the time point ‘2 month’ was exactly the time when the increase of the CA19-9 level showed the strongest mortality risk. This was confirmed by the sequent analysis which showed that patients with CA19-9 ≥ 1000 U/mL had a worse prognosis in the follow-up of 2 and 3 month after adjusting other covariates. The HR for the effect of CA19-9 on OS reached the top at 2 month, and then weakened. (Table 2) And we found that PC patients with pretreatment CA19-9 < 1000 U/mL and 2 month CA19-9 < 1000 U/mL had a longer median survival compared to those with both ≥ 1000 U/mL (8.8 *vs* 5.9 months, *p* < 0.01). Sensitivity analysis based on different cutoff levels showed consistent results. An interesting thing was that the change of the cutoff level didn't affect the survival (median survival: 6 months) of the subgroup with pretreatment CA19-9 ≥ 1000 U/mL whether a 2 month CA19-9 level was larger than 1000 U/mL or not. This might explain why we did not get a statistically significant result in multivariate analyses which adjusted the pretreatment CA19-9 in the model (Table 2). However, for the subgroup with pretreatment CA19-9 < 1000 U/mL, patients with 2 month CA19-9 < 1000 U/mL had a longer median survival compared to those with 2 month CA19-9 ≥ 1000 U/mL. One might conclude that patients with pretreatment CA19-9 ≥ 1000 U/mL unavoidably will have a poor prognosis even though they undergo systemic chemotherapy. Perhaps we should focus more on the patients with pretreatment CA19-9 < 1000 U/mL and take effective measures to maintain a stable CA19-9 level. We cannot discuss the subgroups (1: pretreatment CA19-9 < 1000 U/mL, two months ≥ CA19-9 1000 U/mL; 2: pretreatment CA19-9 ≥ 1000 U/mL, two months CA19-9 < 1000 U/mL) due to the limited sample size.

The main limitations of this investigation arise from its retrospective nature and the consequent missing data distributing unevenly in different variables. Indicators of PC progression could have been superior endpoints to illustrate the influence of pretreatment CA19-9 or CA19-9 kinetics on PC survival compared with OS. However, due to the unavailability of the PC progression information, the relationship between CA19-9 and PC progression could not be discussed. And likewise, some possible prognostic factor such as tumor location, tumor size and pathological characteristics had not be adjusted when performing multivariate analysis because of data constraints. However, since that the effect of CA19-9 on OS was our real concern rather than other possible covariates, it was reported that the Andersen-Gill model gives a nearly unbiased estimate of the treatment effect, even when an important covariate has been omitted. The naive estimate of variance may be too small, but the robust estimate corrects for this by using “sandwich” estimator [29]. Lastly the authors are aware of the fact that the direct clinical applicability of results generated from this novel analytic model may be limited. However, given the lack of researches consisting of both an entire path of CA19-9 monitoring and varying effect of CA19-9 in advanced pancreatic cancer, if validated by a prospective clinical trial in an independent patient cohort, our approach may in fact provide a further step for establishing this biomarker as a useful tool in pancreatic cancer.

In conclusion, this retrospective cohort study constructs an entire path effect variation of CA19-9 kinetics on OS by modeling an extended Cox regression with a time varying coefficient and a time varying covariate. Then the CA19-9 measurement at 2 month is confirmed to better predict the advanced PC patients’ prognosis in combination with the pretreatment CA19-9 measurement. These results suggest that pretreatment CA19-9 and CA19-9 kinetics may serve as a useful serum biomarker to predict patients’ prognosis in advanced pancreatic cancer. But the underlying mechanisms behind this relationship should be explored for possible therapeutic intervention measures.

## MATERIALS AND METHODS

### Study design

We performed a retrospective review in a mega population-based electronic inpatients database. This database was established in 2011 and had kept updating on daily basis ever since, all country-level and above hospitals within Shanghai Metropolitan area, China, which were also qualified for cancer diagnosis, were responsible for tracking and reporting relevant information of all admitted patients, such as specifics of diagnosis, results of tests and examination, and treatment details. 669 historically confirmed PC patients were identified with prior chemotherapy from this database between 1 January 2012 and 31 December 2013. PC patients who underwent a pancreatectomy were excluded (*N*=152), and those without Gemcitabine in their first-line chemotherapy (*N* = 88) or without a pretreatment CA19-9 measurement (*N* = 269) or with a pretreatment CA19-9 measurement < 5 U/mL (*N* = 18) or with a single pretreatment CA19-9 measurement (*N* = 33) were also excluded from the study. Finally, we got 109 PC patients in our study.

The outcome of interest was the OS, which was defined as the time interval between the initiation of chemotherapy and death from any cause. The date of death for PC patients was determined through external matching with the death registration system. The matching deadline was set as 31 January 2015. The whole study was approved by Institutional Research Ethics Board of Fudan University.

### CA19-9 measurement

Due to the fact that the patients were from different hospitals in Shanghai and used different instruments to measure serum CA19-9, method-related differences may exist in results of CA19-9, which was not ideal, given that CA19-9 measurements could vary according to the detection method [42]. However, all the CA19-9 measurements for any given patient were performed at the same laboratory, ensuring some degree of intrapatient consistency. To exclude patients with undetectable CA19-9 (Le^a-b-^ phenotype), pretreatment CA19-9 measurement > 5 U/mL was requested for each included patient. Pretreatment CA19-9 level was defined as the last measurement before the first use of chemotherapeutic drugs. For the analysis of CA19-9 kinetics, serial CA19-9 measurements were not taken on a defined schedule. The frequency of monitoring was directly related to how actively patients were surveyed and depended partly on whether they received maintenance chemotherapy. We retained the first one if there were several times of CA19-9 measurements in one week.

### Statistical analysis

All CA19-9 measurements were treated as a continuous variable and were transformed by taking the logarithm [log_10_(CA19-9)]. The effect of pretreatment CA19-9 levels on OS was modeled by Cox proportional hazard regression and the strength of effect was measured by hazard ratio (HR). The association between CA19-9 kinetics and OS was also modeled by Cox model where CA19-9 measurements were transformed again by taking the logarithm [log_10_ (CA19-9)]. Counting process approach was applied in Cox model to deal with time varying covariates[log_10_ (CA19-9) kinetics], this approach splits every single original observation into a group of “subobservations” at time points when a specific covariate varied. It assumes that the value of this time-varying covariate stays put between two consecutive time points. Thus, within the transformed database, for every “subobservation,” all covariates will be static, and multiple failure-time Cox model under different further assumptions, such as fixed or changed baseline hazard, constant or variant effect along with survival time, can therefore be applied. The major difference between multiple failure-time Cox proportional hazard model and common Cox proportional hazards model is that, the former further adjusts for correlation between “subobservations” stemmed from the same original observation to get a robust variance, by using “sandwich” estimator for instance [43]. Considering that the effect of CA19-9 on OS may change over time, the PH assumption was investigated graphically by a schoenfeld residual plot (Supplementary Figure 1) and the result indicated that the regression coefficient *β*(·) of a time-dependent covariate is a function of the follow-up time. (Supplementary Figure 2) Then a Cox model with a varying-coefficient and a varying-covariate was considered, which could be written as,

$$\lambda (t|\mu_{i},X_{i}(t))=\lambda_{0}(t)exp[\gamma \cdot \mu_{i}+\beta (t)\cdot X_{i}(t)]$$

where **λ (t | μ_i_, X(t))** refers to the instantaneous hazard rate at time point **t** given **μ**_i_ and **X(t)**; **λ_0_ (t)** is the baseline hazard function, **μ_i_** describes the baseline covariates of patient **i**. **X_i_(t)** describes the one-dimensional marker measurement of patient **i** at time **t**. The regression coefficients of the Cox regression for the baseline covariates are given by the vector **γ**, and the coefficient for the effect of the biomarker is quantified by **β(t)**, which is a function of the follow-up time. The function **β(t)** is obtained by estimating **β** at numerous intervals in time and then smoothing over these pointwise estimates using natural cubic spline functions. OS was displayed using Kaplan-Meier survival curves and the differences between subgroups were compared using the log-rank test. We checked the assumptions of Cox regression models by using graphically based residual analyses where we evaluated the proportional hazards assumption, the functional form of the continuous covariates and possible influential observations. These analyses were performed with SAS (version 9.3, SAS Institute Inc., Cary, NC,) and R (version 3.0.2), and a probability *P* value of 0.05 or lower was considered statistically significant.

## SUPPLEMENTARY MATERIALS FIGURES



## Abbreviations

PC: pancreatic cancer
OS: overall survival
CA19-9: carbohydrate antigen 19-9
PH: proportional hazard
HR: hazard ratio
