# Supplementary material for: A varying-coefficient cox model for the effect of CA19-9 kinetics on overall survival in patients with advanced pancreatic cancer
Source: Oncotarget. 2017 Feb 21;8(18):29925–34. doi: 10.18632/oncotarget.15557 (PMC5444714; doi:10.18632/oncotarget.15557)
Supplement: Supplementary file 1 [file oncotarget-08-29925-s001.pdf]

A varying-coefficient cox model for the effect of CA19-9 kinetics on overall survival in patients with advanced pancreatic cancer

SUPPLEMENTARY FIGURES

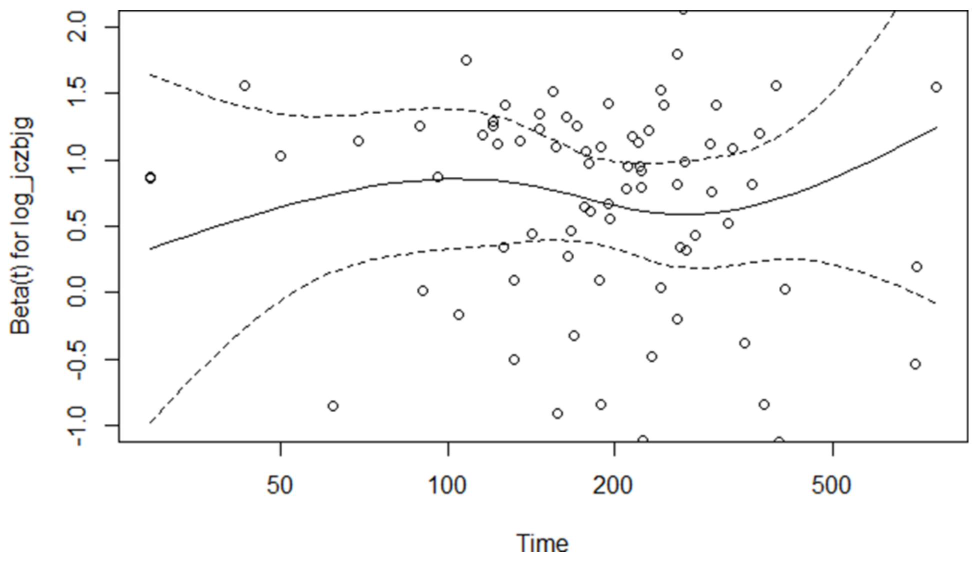

Supplementary Figure 1: The schoenfeld residual plot for CA19-9 kinetics, test of proportional hazard.

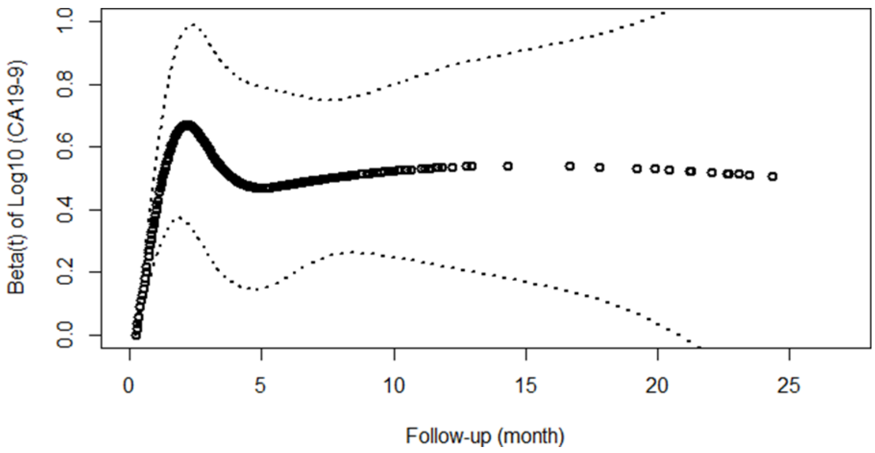

Supplementary Figure 2: The coefficient's trajectory over time that corresponds to CA19.9 kinetics along with its time dependent CIs.
